# Part II: The Influence of Crosslinking Agents on the Properties and Colon-Targeted Drug Delivery Efficacy of Dextran-Based Hydrogels

**DOI:** 10.3390/gels12010025

**Published:** 2025-12-28

**Authors:** Tamara Erceg, Miloš Radosavljević, Milorad Miljić, Aleksandra Cvetanović Kljakić, Sebastian Baloš, Katarina Mišković Špoljarić, Ivan Ćorić, Ljubica Glavaš-Obrovac, Aleksandra Torbica

**Affiliations:** 1 Faculty of Technology Novi Sad, University of Novi Sad, Bulevar cara Lazara 1, 21000 Novi Sad, Serbia; milosr@tf.uns.ac.rs (M.R.); a.c.istrazivac@gmail.com (A.C.K.); 2 Institute of Food Technology in Novi Sad, University of Novi Sad, Bulevar cara Lazara 1, 21000 Novi Sad, Serbia; milorad.miljic@fins.uns.ac.rs (M.M.); aleksandra.torbica@fins.uns.ac.rs (A.T.); 3 Faculty of Technical Sciences, University of Novi Sad, Trg Dositeja Obradovića 6, 21000 Novi Sad, Serbia; sebab@uns.ac.rs; 4 Faculty of Medicine, Josip Juraj Strossmayer University of Osijek, Josipa Huttlera 4, 31000 Osijek, Croatia; kmiskovic@mefos.hr (K.M.Š.); icoric@mefos.hr (I.Ć.); lgobrovac@mefos.hr (L.G.-O.)

**Keywords:** dextran-based hydrogels, divinyl benzene, diethylene glycol diacrylate, 4,4′-di(methacryloylamino)azobenzene, colon-targeted drug delivery

## Abstract

In this study, dextran-based hydrogels were synthesized in dimethyl sulfoxide via free-radical polymerization with three structurally different crosslinking agents: divinyl benzene (DVB), diethylene glycol diacrylate (DEGDA), and 4,4′-di(methacryloylamino)azobenzene (DMAAazoB). Their morphology, swelling ability, mechanical properties, and potential for controlled release of the model substance (uracil) were examined, with the results showing that the chemical structure and chain length of the crosslinking agents significantly influence the structural and functional properties of hydrogels. Hydrogels crosslinked with DMAAazoB showed the highest swelling ability at pH 3 and pH 6 (2552 and 1696%, respectively), associated with protonation effects and sponge-like morphology, while simultaneously showing the lowest mechanical strength (20 and 47 MPa). In vitro simulations of gastrointestinal digestion showed that uracil was not released in the gastric phase, while in the intestinal environment, the release was significant, especially in Dex-DMAAzoB hydrogels (88.52%). The absence of azoreductases in the simulated system indicates that the release of the drug in real conditions would likely be even more pronounced. The Dex-DAAazoB hydrogel exhibited a slight antibacterial effect, producing inhibition zones of 8 and 7 mm against *Escherichia coli* ATCC 8739 and *Staphylococcus epidermidis* ATCC 12228, respectively. In contrast, the remaining hydrogel formulations showed no detectable antibacterial activity toward either bacterial strain, indicating their microbiological inertness and supporting their suitability as carrier matrices for antitumor drug delivery in colorectal cancer therapy. The obtained results confirm that azo-crosslinked dextran hydrogels, with an optimized amount of crosslinking agent, are promising carriers for the targeted and controlled delivery of antitumor drugs to the colorectal region.

## 1. Introduction

Colorectal cancer is the third most common form of cancer and the second most common cause of cancer deaths globally. According to the latest data from the International Agency for Research on Cancer (IARC), about 1.9 million new cases of this type of cancer are diagnosed worldwide every year, with over 900,000 mortalities [1]. Targeted chemotherapy significantly advances the treatment of colorectal cancer (CRC) by targeting specific molecular abnormalities within cancer cells, resulting in greater efficacy, reduced side effects, and improved patient outcomes compared to traditional chemotherapy regimens. Biopolymer carriers for antitumor drugs play a key role in improving the delivery of targeted therapies for colorectal cancer (CRC), providing numerous advantages such as biocompatibility, biodegradability, and more effective tumor-site targeting [2]. Carriers based primarily on polysaccharides enable controlled, sustained drug release, helping maintain effective therapeutic levels at the tumor site while reducing harm to surrounding healthy tissues. Their use can also support oral administration by shielding drugs from premature degradation. A particularly promising strategy for achieving colon-targeted delivery is polymer systems that are specifically degraded by colonic bacteria; the colon hosts abundant polysaccharide enzymes, which can be harnessed to trigger site-specific drug release. Following this principle, numerous polysaccharides—such as chitosan, pectin, chondroitin sulfate, alginate, guar gum, hyaluronic acid, ethyl cellulose, inulin, and dextran—have been explored as carriers for colon-specific drug delivery. The numerous functional groups present in these polysaccharides enable diverse chemical modifications, allowing fine-tuning of their physicochemical properties, degradation profiles, and drug-release behavior [3]. A wide range of polysaccharide-based systems have been investigated for colon-specific drug delivery, formulated in various oral dosage forms designed to withstand the upper gastrointestinal tract and undergo degradation primarily in the colon [4]. Of these, enteric-coated chitosan capsules [5], as well as matrices prepared from chitosan succinate and chitosan phthalate [6], have demonstrated promising pH- and enzyme-responsive behavior. Pectin-based systems, including pectin salt matrices [7] and amidated pectin matrix tablets [8], have also been extensively studied due to their susceptibility to colonic pectinolytic enzymes. Further improvements have been achieved using amidated pectin/calcium pectinate matrix tablets, often combined with ethyl cellulose [9] as a release-modifying additive to enhance site specificity and control drug liberation. Other polysaccharides explored for this purpose include chondroitin sulfate, which has been used to formulate crosslinked chondroitin matrix tablets to improve mechanical stability and enzyme-triggered degradation [10]. Additionally, calcium alginate beads, particularly double-coated variants for providing swellability and delaying permeability, have shown potential for delivering active compounds selectively to the colon [11]. Guar gum-based matrix tablets have shown promising performance for colon-targeted delivery by minimizing premature drug release in the upper GIT and promoting controlled release upon exposure to colonic fluids [12]. Ethylcellulose-based tablets have shown excellent potential for modulating drug release profiles, enabling controlled and colon-specific delivery [13]. Inulin tablets exhibit minimal drug release in the upper gastrointestinal tract (GIT) and provide colon-specific release triggered by bacterial enzymatic activity [14]. Dextran-based capsules and tablets have demonstrated limited drug release in the upper GIT, while drug liberation markedly increased in the presence of rat caecal contents in pH 6.8 buffer, confirming the susceptibility of dextran matrices to colonic enzymatic degradation [15]. In addition to capsules and tablets, other oral dosage forms based on polysaccharides—such as microspheres, nanoparticles, microparticles, and hydrogels [16]—have demonstrated the ability to deliver active compounds to the colorectal region in a controlled manner. These systems employ various encapsulation strategies to incorporate antitumor agents for colorectal cancer therapy, utilizing polysaccharides as carriers to modulate drug release profiles. Traditional approaches for achieving colon-targeted drug delivery generally rely on four main concepts: (1) prodrug/azo-polymer-based systems, (2) pH- and time-dependent systems, (3) pressure-sensitive systems, and (4) gut microflora-activated systems [17,18]. Among these polysaccharide-based carriers, dextran-based hydrogels offer distinct advantages. As three-dimensional polymer networks, they can efficiently encapsulate antitumor drugs and provide a versatile platform for integrating multiple targeting mechanisms. This dual functionality enhances the probability of delivering drugs specifically to the colorectal region while minimizing systemic side effects [19].

Dextran-based hydrogels, in particular, are promising carriers for colon-targeted drug delivery because they are selectively biodegraded by colonic enzymes, such as dextranase and amylase, ensuring site-specific release of the encapsulated drug. The incorporation of azo-based crosslinkers further enables enzymatic cleavage by microbial azoreductases, thereby synergistically enhancing site-specific drug release and increasing local drug concentrations within the colon. Various strategies have been reported for the preparation of dextran-based hydrogels, employing distinct crosslinking mechanisms. Some approaches rely on addition reactions, using crosslinkers such as hexamethylene diisocyanate [20] and epichlorohydrin [21] without prior dextran modification, whereas others utilize free-radical polymerization, enabled through the preliminary methacrylation of dextran [22,23]. In our previous work, “Part I” [24], we described an energy-efficient process for the synthesis of hydrogels based on dextran and inulin in an aqueous medium at room temperature, beginning with modified biopolymers that were crosslinked using free-radical polymerization. In this way, we established the possibility of incorporating thermolabile components in situ, which is a significant advantage in the development of carriers for biomedical applications. Based on the results of our digestion and swelling studies, we concluded that further research should focus on dextran-based hydrogels, as they achieved higher drug release in intestinal fluid (over 60%) compared to hydrogels formulated with added inulin. Accordingly, in this work, we analyzed the influence of different crosslinkers on the structural and functional properties of dextran hydrogels. Special attention was paid to their influence on network morphology and their susceptibility to enzymatic degradation, which are key parameters for the optimization of carriers in biomedical applications.

Despite the extensive research into dextran-based hydrogels, no systematic study has addressed their synthesis through a simple crosslinking strategy employing structurally distinct crosslinkers such as divinylbenzene, diethylene glycol diacrylate, and 4,4′-di(methacryloylamino)azobenzene. The present work addresses this gap by evaluating how these crosslinkers, applied under identical synthesis conditions, affect the structural and mechanical properties, swelling behavior, and colon-targeted drug delivery performance of dextran hydrogels. This comparative approach provides novel insights into the rational design of dextran-based hydrogels as advanced carriers for site-specific therapeutic applications. Additionally, we present a simplified two-step synthesis method and the energy-efficient transformation of the hydrogels into a granulated state, enabling direct application in oral dosage forms such as capsules. By combining structural versatility with practical formulation strategies, this work offers a unique framework for developing highly adaptable, colon-targeted hydrogel systems.

## 2. Results and Discussion

### 2.1. Results of FTIR Analysis

**Figure 1:**
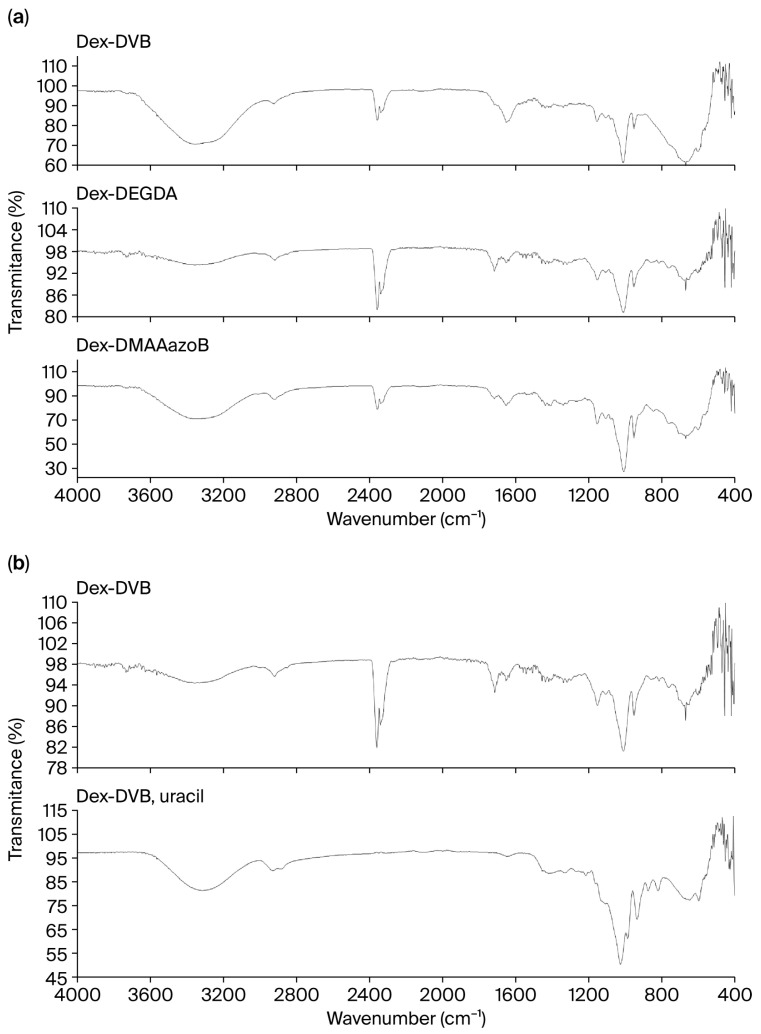
FTIR spectra of (**a**) dextran-based hydrogel synthesized using DVB, DEGDA, and DMAAazoB as crosslinking agents and (**b**) Dex-DVB and uracile-loaded Dex-DVB hydrogel.

The prepared hydrogels exhibited very similar FTIR spectra, with all samples demonstrating a broad band at 3300 cm^−1,^ which corresponded to the stretching vibration of the OH group, two peaks between 2930 and 2800 cm^−1^ assigned to the -CH stretching vibrations, and an absorption band at 1721–1715 cm^−1^ attributed to the carbonyl group (Figure 1). The FTIR spectrum of neat dextran is given in the Supplementary Material (Figure S1). In all spectra, two peaks between 1420 and 1335 cm^−1^ represent the bending modes of CH_2_ and CH_3_, the peak at 1154–1157 cm^−1^ corresponds to the C-O-C stretching vibration, a small peak at 1045 cm^−1^ represents the S=O group from DMSO, the absorption bands in the 1080–1015 cm^−1^ region represent C-C-O stretching and C-O-H, and the peaks at 915–800 cm^−1^ are attributed to the glycosidic bond. Also, in the FTIR spectrum of Dex-DMAAazoB, there is a small peak at 3009 cm^−1^, attributed to the C-H band in the benzene ring of the crosslinking agent (DMAAazoB). Figure 1b compares the FTIR spectra of neat Dex-DVB and the uracil-loaded gel; no new absorption bands that could be unambiguously attributed to uracil were observed. This is most likely due to the overlap of the characteristic vibrations of uracil (C=O and ring modes) with the broad OH and C–O signals of the dextran matrix. Only minor changes were detected in the region between 1700 and 1650 cm^−1^. The FTIR spectrum of uracil is given in the Supplementary Material (Figure S2).

### 2.2. Results of SEM Analysis

**Figure 2:**
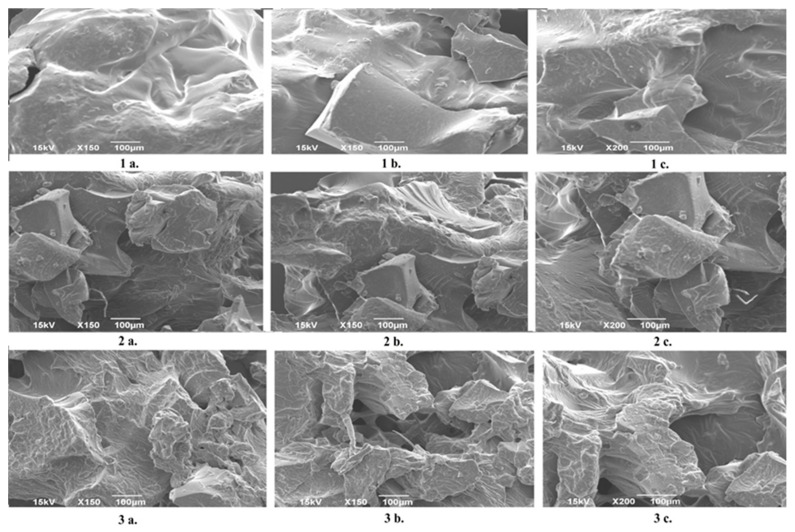
SEM images of hydrogels at different magnifications (100 and 200×): Dex-DEGDA (**1a**–**1c**); Dex-DVB (**2a**–**2c**); Dex-DMAAazoB (**3a**–**3c**).

Figure 2 displays the SEM images of dextran-based hydrogels prepared with different crosslinking agents. Although the same quantity was applied for each crosslinker, their distinct chemical structures resulted in markedly different morphologies, consistent with the macroscopic appearance of the materials. The hydrogel crosslinked with DEGDA exhibits smooth, compact morphology and more regular fracture angles after granulation, reflecting its more flexible and uniform network. In contrast, the Dex–DVB hydrogel is noticeably more brittle, which can be attributed to the rigid aromatic ring and short chain length of the DVB crosslinker, and the Dex–DMAAazoB hydrogel displays a sponge-like structure, characterized by collapsed pores, likely due to the long, flexible chain of the azo-based crosslinker. The SEM analysis reveals a rough, irregular surface. Compared with the dextran hydrogels synthesized in aqueous media at room temperature in our previous work [24], the morphology of the present hydrogels shows certain differences. This is particularly evident for the DMAAazoB-crosslinked system, which appears more elastic and exhibits the highest resistance to shredding in its dried state.

**Figure 3:**
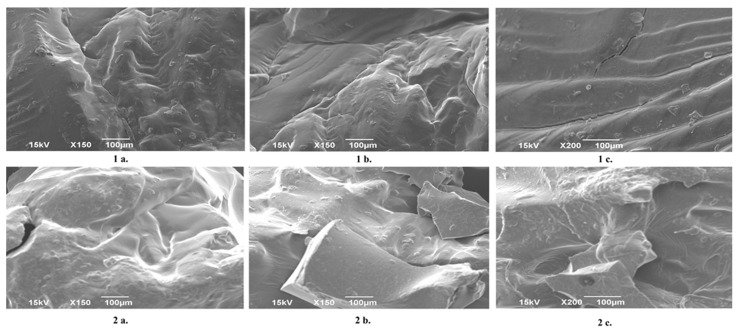
SEM images of neat Dex-DEGDA hydrogel (**1a**–**1c**) and uracil-loaded Dex-DEGDA hydrogel (**2a**–**2c**).

Figure 3 compares the morphology of a neat Dex–DEGDA hydrogel with that of a uracil-loaded Dex–DEGDA sample. The incorporation of uracil from solution did not give rise to any visible structural features in the SEM images, rendering its presence indistinguishable by this technique.

### 2.3. Results of Swelling Analysis

**Figure 4:**
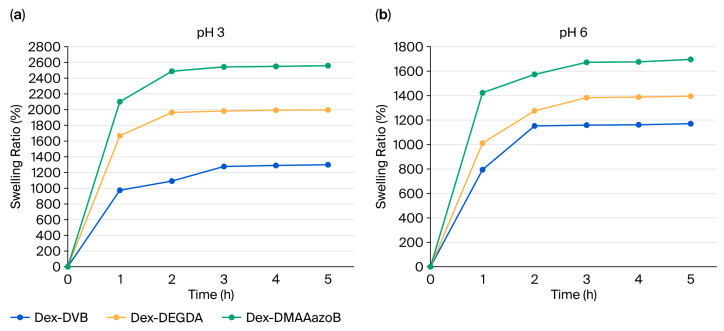
Swelling curves of hydrogels at (**a**) pH 3 and (**b**) pH 6.

Figure 4 illustrates the swelling capacity of hydrogels synthesized with three different crosslinking agents at pH 3 and 6. The swelling ability of hydrogels at both pH values increases with the length of the crosslinking agent. DEGDA, having a longer and more flexible chain compared to DVB, creates a polymer network with a larger chain distance, which translates into the higher swelling capacity of hydrogels crosslinked with DEGDA. At both pH values, hydrogels with the same structural composition generally show similar trends and comparable swelling abilities. A notable exception is the hydrogel synthesized with the addition of 4,4-di(methacryloylamino)azobenzene (azo). This difference arises because azo compounds, especially those with amino substituents, can undergo protonation of their azo and amino groups in acidic environments. At pH 3 (acidic), the protonation of these groups in a Dex-DMMAazoB hydrogel introduces positive charges within the polymer network [25,26]. This leads to increased electrostatic repulsion between the polymer chains, forcing the network to expand and resulting in a significantly higher swelling ability at this lower pH compared to other hydrogels or their behavior at higher pHs. The Dex-DMMAazoB hydrogel shows the highest swelling capacity at both pH 3 and pH 6 compared to other formulations. This superior performance is likely due to its sponge-like morphology, which is in accordance with the results of our SEM analysis. The statistical analyses are given in the Supplementary Material (Table S1a,b).

### 2.4. Results of Mechanical Property Analysis

**Figure 5:**
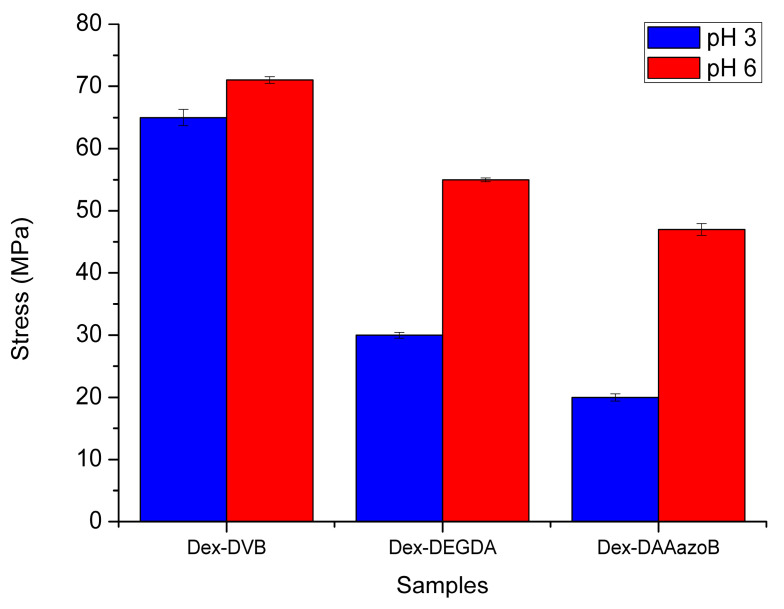
Granulated hydrogel stress (elastic moduli) values at pH 3 and pH 6 in the equilibrium swelling condition.

As a consequence of the inverse relationship between swelling ability and mechanical strength, hydrogels showed higher mechanical strength at pH 6 than at pH 3, which is reflected in the higher elastic modulus values (Figure 5). The hydrogel synthesized with DMMAzoB had the lowest shear modulus, indicating the lowest stiffness among all formulations. The statistical analyses are given in the Supplementary Material (Table S2a,b). The shear moduli of the hydrogels described in this study exhibit similar values to those reported in our previous work [24], consistent with the observed swelling behavior and the comparable crosslinking densities of the hydrogels. The statistical analyses are given in the Supplementary Material (Table S2a,b), and average values with standard error are presented in Figure 5.

### 2.5. Results of In Vitro Gastrointestinal Digestion (GID)

The results of the digestion process, as expressed through the activity of enzymes originating from different segments of the digestive tract, are presented in Table 1, which summarizes the average values along with their standard deviations. Measurements were carried out in triplicate, and statistical analysis is given in the Supplementary Material (Table S3). During the gastric phase, the carrier did not release uracil, while a significant release was recorded in the intestinal environment, with the hydrogel synthesized with an azo-crosslinker (Dex-DAAazoB) showing the most pronounced. Considering that the applied INFOGEST matrix does not contain azoreductases, it is likely that the release would be even more extensive in their presence in vivo. The dextran-based hydrogels synthesized in this study, relying solely on dextran and their respective crosslinking agents, showed no susceptibility to hydrolysis in gastric fluid, resulting in the absence of uracil release in this phase. Consequently, the amount of uracil released in the intestinal phase is lower than for the hydrogels prepared in our previous research [24], except for Dex-DMAAazoB, which is higher. This behavior is likely associated with the susceptibility of the azo crosslinking bond, as well as the overall network architecture, including the accessibility of glycosidic linkages to enzymatic activity [27]. These findings highlight that both the chemical composition and the three-dimensional network structure of hydrogels play crucial roles in their degradation and, consequently, in the release profile of the encapsulated compound. The results obtained indicate that the structural characteristics and crosslinking chemistry of the Dex-DMAAazoB gel make it the most promising candidate for controlled targeted drug delivery in the colorectal region. The statistical analyses are given in the Supplementary Material (Table S3). 

### 2.6. Determination of Antimicrobial Activity

The antimicrobial behavior of neat dextran-based hydrogels provides valuable indirect insight into their biocompatibility and potential cytotoxicity. Although dextran itself is a biocompatible, non-toxic polysaccharide widely used in biomedical applications, the introduction of different crosslinking agents can alter the physicochemical properties of the network and, consequently, its interaction with microorganisms and mammalian cells [28]. Taking this into account, the susceptibility of *Escherichia coli* ATCC 8739 (Gram-negative) and *Staphylococcus epidermidis* ATCC 12228 (Gram-positive) to dextran is typically greater than that of mammalian cells; as such, we investigated the potential antibacterial activity of the three dextran-based hydrogels against these microorganisms. Differences in susceptibility arise from fundamental biological and structural features that distinguish prokaryotic microorganisms from eukaryotic cells. Bacteria possess a cell wall composed of peptidoglycan layers and, in Gram-negative species, an additional outer membrane, which makes them considerably more vulnerable to disruption by reactive compounds, osmotic stress, or membrane-active substances. In contrast, mammalian cells have more stable and tightly regulated plasma membranes, together with advanced intracellular repair mechanisms. Furthermore, bacteria exhibit limited capacity to adapt to stress and therefore rapidly lose viability when exposed to unfavorable physicochemical conditions, whereas mammalian cells often develop enhanced survival strategies, including increased resistance to oxidative stress, evasion of apoptosis, and more efficient damage-repair pathways. Consequently, many materials—including polymers, nanoparticles, and various crosslinking agents—display significant antimicrobial activity at concentrations that are not harmful to mammalian cells, meaning that bacteria are inhibited or destroyed at far lower exposure levels. Thus, if neat hydrogels do not exhibit antimicrobial activity, it strongly suggests that they also do not possess cytotoxic effects toward mammalian cells, although dedicated cytotoxicity assays are still required for confirmation [29].

**Figure 6:**
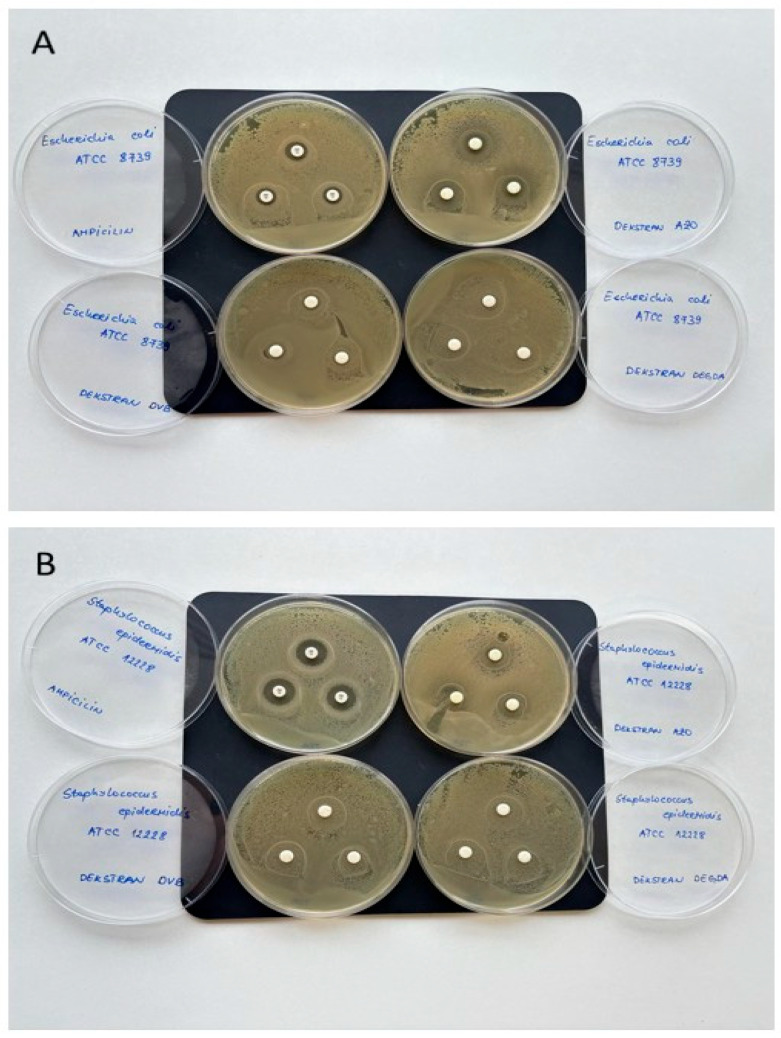
Antimicrobial activity of dextran-based samples against *Escherichia coli* ATCC 8739 (**A**) and *Staphylococcus epidermidis* ATCC 12228 (**B**).

Antimicrobial activity was assessed by measuring the diameter of the inhibition zone (Figure 6), and the results are summarized in Table 2. Data showed that Dex-DAAazoB had a slight inhibitory effect on both tested bacterial strains. On the other hand, it was found that other dextran-based samples did not exhibit an inhibitory effect on *E. coli* and *S. epidermidis*; therefore, their application as carriers for the antitumor drugs in colorectal cancer therapy is justified.

## 3. Conclusions

Our previous study demonstrated the ability of dextran-based hydrogels to act as carriers for colon-targeted drug delivery; in this study, they were successfully synthesized via free-radical polymerization with three structurally different crosslinking agents: divinylbenzene (DVB), diethylene glycol diacrylate (DEGDA), and 4,4-di(methacryloylamino)azobenzene (DMAAazoB). The obtained results confirmed that the chemical nature and length of the crosslinker chain have a key influence on the morphology, swelling ability, mechanical properties, and drug release efficiency of the synthesized hydrogels. Hydrogels prepared with DMAAazoB showed the highest degree of swelling at both tested pH values (2552 and 1696%, respectively), which can be explained by their protonation effects and sponge-like morphology. However, these hydrogels also showed the lowest mechanical strength (20 and 47 MPa), which is consistent with the inverse relationship between swelling and network rigidity. The results of in vitro gastrointestinal digestion indicated that uracil was not released from the hydrogels in the gastric phase, while a pronounced release occurred in the intestinal environment, in which the Dex-DMAAazoB hydrogels achieved the highest level of efficiency (88% uracil released in the intestinal phase). The antimicrobial assay revealed that Dex-DMAAzoB exhibited a slight inhibitory effect against both tested bacterial strains, whereas the other two hydrogels showed no inhibition toward *E. coli* or *S. epidermidis*. Such behavior indicates that the prepared hydrogels are suitable for use as carriers, as they do not display undesired antimicrobial activity that could imply potential cytotoxicity.

Although azoreductases were not included in the simulation, it is likely that their presence would further enhance drug release from azo-crosslinked structures. Therefore, the obtained results confirm that azo-crosslinked dextran hydrogels are promising carriers for targeted and controlled delivery of antitumor drugs to the colorectal region. These findings provide significant guidelines for the rational design and optimization of biodegradable hydrogels intended for use in targeted cancer therapy.

## 4. Materials and Methods

### 4.1. Materials

Dextran from Leuconostoc (M_r_ = 40,000), glycidyl methacrylate (GMA), divinyl benzene (DVB), initiator azobisobutyronitrile (AIBN), and diethylene glycol diacrylate (DEGDA), as well as the digestion enzymes and agents—pepsin, dextranase, pancreatin, bile salts, Pefabloc^®^, CaCl_2_ · 2H_2_O, MgCl_2_·6H_2_O, (NH_4_)_2_CO_3_, KCl, KH_2_PO_4_, NaCl, and NaHCO_3_—were procured from Sigma Aldrich (St. Louis, MO, USA). Ethanol (96%) was purchased from CENTROHEM (Stara Pazova, Serbia). Dimethyl sulfoxide (DMSO) was supplied by Merck KGaA (Darmstadt, Germany). 4-dimethylaminopiridine (4-DMAP) was provided by Thermo Fisher Scientific Inc. (Waltham, MA, USA), while acetone was obtained from T.T.T. d.o.o. Sveta Nedelja (Sveta Nedelja, Croatia). 4,4-di(methacryloylamino)azobenzene (DMAAazoB) was supplied by Amadis Chemical Company Limited (Hangzhou, Zhejiang Province, China). Buffer solutions with pH values of 3 and 6 were purchased from Reagecon (Shannon, Ireland).

### 4.2. Preparation of Hydrogels

**Figure 7:**
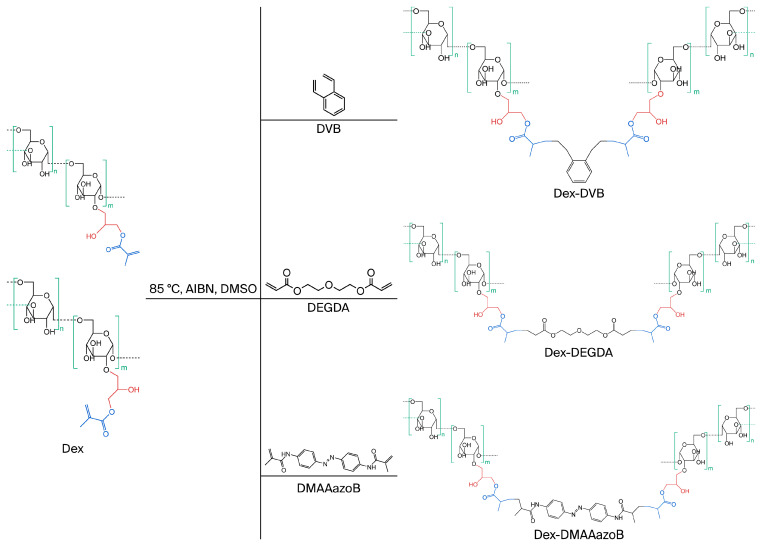
The reaction between the functional groups of modified dextran (Dex) and the crosslinking agents: DVB, DEGDA, and DMAAazoB.

**Figure 8:**
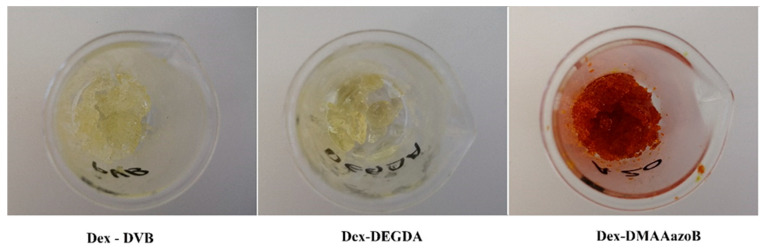
Visual appearance of prepared hydrogels.

Dextran was modified according to the procedure described in “Part I” [24]. The dextran modification process was initiated by dissolving it in DMSO at a concentration of 10 wt% at a temperature of 50 °C with continuous stirring. When the biopolymer was completely dissolved, the DMAP catalyst was added (7 wt% relative to dextran). After the catalyst was completely dissolved, the solution was cooled to room temperature. Glycidyl methacrylate (GMA) was then added to the mixture at a weight ratio of 3:2 relative to the biopolymer. The modification reaction proceeded for 18 h at room temperature and then stopped by adding acetone, which was used to precipitate the modified dextran. As demonstrated in our previous study (“Part I”) [24], FTIR analysis confirmed the successful modification of dextran, which exhibited a methacrylation degree of 1.2 functional groups per monomer unit. Next, 1 g of modified dextran (dextran-methacrylate, Dex-MA) was dissolved in 5 mL of DMSO at 50 °C. After complete dissolution of Dex-MA, 0.1 g of a certain crosslinking agent—either DVB or DEGDA—was added to the dissolved Dex-MA, and after increasing the temperature to 85–90 °C, 0.07 g of the initiator AIBN dissolved in 1 mL of DMSO was added to the mixture and reacted for 20 min until the gel point was reached. The procedure for preparing hydrogels with DMAAazoB was carried out with one exception due to the nature of the crosslinking agent (powder, not liquid); namely, before adding it to the warmed Dex-MA solution, DMAAazoB was dissolved in 3 mL of DMSO. The other steps followed the procedure for hydrogel preparation with DVB and DEGDA. Figure 7 illustrates the reaction between the functional groups of modified dextran (Dex) and the crosslinking agents, which proceeds via a free-radical polymerization mechanism as follows. The thermal initiator AIBN undergoes homolytic cleavage upon heating (~85–90 °C), generating highly reactive free radicals that can initiate polymerization by attacking the methacrylate double bonds on Dex-MA. This reaction produces polymer-centered radicals, which serve as active sites for chain growth. During the propagation stage, these radicals sequentially react with additional methacrylate groups on Dex-MA or on multifunctional crosslinkers such as DVB, DEGDA, or DMAAazoB, resulting in chain elongation. The multifunctional nature of these crosslinkers enables the formation of covalent bonds between distinct polymer chains, leading to the establishment of a three-dimensional crosslinked network. The gel point is reached when the network becomes continuous throughout the reaction mixture, resulting in a hydrogel that swells in aqueous media but remains insoluble. The choice and structure of the crosslinker (e.g., liquid versus solid) critically influence radical transfer kinetics, network density, and overall hydrogel architecture, which in turn dictate swelling behavior, mechanical strength, and drug release profile. Additionally, one series of hydrogels was prepared by incorporating uracil in situ to explore the potential for drug loading during the polymerization process. Figure 8 demonstrates the visual appearance of the obtained hydrogels. The drug was added before the initiator in accordance with the established procedure. The initial composition of the hydrogels is summarized in Table 3.

### 4.3. Infrared Spectroscopy with Fourier Transformation (FTIR) Analysis

The hydrogel composition was examined using a Shimadzu IRaffinity-1s Fourier Transform Infrared Spectrometer (Shimadzu Corporation, Kyoto, Japan) in Attenuated Total Reflectance (ATR) mode, employing a MIRacle 10 ATR-FTIR; Dia/ZnSe to analyze the structure of modified biopolymers and hydrogels. A total of 40 scans were averaged at a spectral resolution of 4 cm^−1^.

### 4.4. Scanning Electron Microscopy (SEM) Analysis

The structure of the hydrogels was examined using a JEOL JSM-6390 scanning electron microscope (JEOL Ltd., Tokyo, Japan). Before imaging, a conductive coating was applied to the samples with a BALTEC SCD 005 apparatus. The images were captured at magnifications of 150× and 200×.

### 4.5. Analysis of Swelling Properties

The capacity of the hydrogels to swell was examined at the physiological temperature of 37 °C in buffer solutions with pH levels of 3 and 6 over a duration of 5 h. This method was designed to replicate the environment of the digestive tract. The equilibrium swelling degree (ESD) was calculated using Equation (1):

(1) $$ESD\;(\%)=\;\frac{m_{t}-m_{0}}{m_{0}}\cdot 100\%$$

where *m_t_* represents the weight of the swollen hydrogel at various time points (1, 2, 3, 4, or 5 h), and *m*_0_ is the initial weight of the dry hydrogel. Measurements were performed in triplicate, and results are presented as the mean value.

### 4.6. Analysis of Mechanical Properties

Mechanical properties were determined using the same procedure as in our previous work [24]; 0.3 g of xerogel was weighed and placed in pH 3 and 6 buffers for 5 h at 37 °C. After reaching their maximum swelling capacity, the hydrogel granules were gently extracted from the buffer solutions using a colander to eliminate excess water and were promptly subjected to mechanical characterization. This process involved the application of orthogonal shear stress through a specialized analyzer equipped with a cylindrical probe made of stainless steel that had specific roughness characteristics; the bottom plate measured 25 mm in diameter, with metrics of Ra = 0.371, Rq = 0.485, Rz = 2.787, and Rp = 0.645 µm, while the supporting ring’s metrics were Ra = 0.075, Rq = 0.105, Rz = 0.609, and Rp = 0.381. The roughness metrics were assessed using a Mitutoyo SJ-220 portable surface tester (Mitutoyo Corporation, Kawasaki, Japan). Shear stress was applied incrementally until the hydrogel disintegrated, indicated by the rapid release of trapped water. This technique enabled the evaluation of the hydrogel’s mechanical integrity and its resistance to shear forces while it was in the equilibrium swollen state. Measurements were performed in triplicate, and results are presented as mean ± standard error.

### 4.7. Simulated In Vitro Digestion and Determination of Uracil

Simulated in vitro digestion and determination of uracil were performed according to the procedure described in “Part I” [24]. Standardized static in vitro digestion was carried out in accordance with the protocol described by Kostić et al. (2021) [30], with minor changes in the simulated intestinal phase. Since it was assumed that the hydrogels would be applied and incorporated in capsules, analysis of the oral phase was omitted. The experiment was performed in three independent groups, each in three repetitions. Digestion was initiated in the gastric phase by mixing 0.25 g of the dried hydrogel incorporated into gelatine capsules with 7.5 mL of simulated gastric fluid (SGF), 1.6 mL of pepsin solution (25,000 U/mL), and 0.5 mL of 0.3 M CaCl_2_. The pH value of the mixture was adjusted to 2.0 through the addition of 1 M HCl, and the total volume was brought to 20 mL with distilled water. The samples were incubated at 37 °C for 2 h with constant mixing (250 rpm). In the next (intestinal) phase, the obtained gastric chyme was mixed with 11 mL of simulated intestinal fluid (SIF), 5 mL of pancreatin solution (800 U/mL, trypsin activity), 0.2 mL of dextranase (100 U/mL), 2.5 mL of bile salt solution (160 mM), and 0.4 mL of 0.3 M CaCl_2_. The pH was adjusted to 7.0, and the total volume was brought to 40 mL with distilled water. The samples were incubated for 2 h at 37 °C with continuous mixing (250 rpm). The composition of SGF and SIF is the same as in “Part I”, and is shown in Table S3 of the Supplementary Material. After digestion ended, the enzymatic reactions were stopped by the addition of Pefabloc^®^. Three experimental groups were prepared:

1. Digestive hydrogels with uracil—treated with the specified protocol.
2. Empty hydrogels (control)—immediately mixed with all enzymes and solutions needed for digestion, with the reactions instantly inhibited by the addition of Pefabloc^®^.
3. Digestive cocktail (control)—2.5 g of distilled water instead of the sample, mixed with all enzymes and solutions, with the addition of Pefabloc^®^.

At the end of the treatment, the samples were centrifuged at 10,000 rpm for 20 min at 4 °C. The obtained supernatants were separated, their volume precisely measured, and samples stored at –20 °C until further analysis.

The UV/VIS spectrophotometry method for uracil determination was modified from Khajehsharifi and Soleimanzadegan (2013) [31]. To determine the UV–Vis spectra, an Agilent BioTek EPOCH 2 photodiode spectrophotometer (UV–Vis spectrophotometer (Agilent Technologies, Santa Clara, CA, USA)) equipped with GEN5 software (version 3.11; Agilent Technologies, Santa Clara, CA, USA) and a computer incorporating a quartz cell with an optical path length of 1 cm was used. A concentration of approximately 25 mg sample/25 mL of distilled water was sonicated for 5 min, after which the volume was adjusted to 100 mg/mL. Based on this solution, a series of standards was prepared for the construction of the calibration curve, where the obtained concentrations were 100, 50, 25, 12.5, 6.25, and 3.125 mg/mL. The linear dynamic range of each component was determined by regression analysis, measuring the dependence of absorbance on the corresponding λ_max_ in relation to the concentration of the analyte. For wavelength selection in uracil quantification, the stock standard solution (100 mg/mL) was scanned in the UV range 200–400 nm. The maximum absorbance (λ_max_) was registered at 266 nm, and distilled water was used as the reference solution. For the analysis of the digestate samples, the absorbance was measured at 230 nm, and the digestate containing empty hydrogels, specifically prepared for each tested mixture, was used as a blank.

### 4.8. Determination of Antimicrobial Properties of Neat Hydrogels—Disc Diffusion Method

In order to evaluate the antimicrobial activity of the dextran-based samples, the Gram-negative bacteria Escherichia coli ATCC 8739 and the Gram-positive bacteria Staphylococcus epidermidis ATCC 12228 were used. These strains were chosen because they are common causes of bacterial infections. Reference strains were purchased lyophilized from the American Type Culture Collection (Manassas, VA, USA) and kept in the culture collections at the Institute of Food Technology, University of Novi Sad. They were stored at −80 °C in Trypto-Casein Soy Broth (TSB; Biokar BK046 HA, Beauvais, France) supplemented with 15% glycerol and revitalized by cultivation on tryptic soy agar (TSA; Oxoid CM0131, Hampshire, UK) for 24 h at 37 °C before experiments. For the antimicrobial activity assays, a loop of actively growing cells from each strain was suspended in phosphate-buffered saline (PBS; Oxoid, Hampshire, UK, pH 7.3) and adjusted to match the turbidity of a 0.5 McFarland standard. Next, bacterial suspensions were diluted in TSB to achieve a final cell concentration of 1 × 10^6^ colony-forming units (CFU) per mL (CFU/mL). The disc diffusion method was used to study the antibacterial activity of three samples based on dextran, as previously reported by Šuput et al. (2024) [32], with some modifications. Tests were also conducted with ampicillin (Bioanalyse, Ankara, Turkey) for bacteria as a control for microorganism sensitivity. Briefly, 100 µL of the prepared cell suspension (1 × 10^6^ CFU/mL) was evenly spread across a TSA agar plate, and then sterile filter paper discs (about 6 mm in diameter) were placed on the agar surface. To measure the effect on bacterial growth, the discs were inoculated with 10 µL of a stock solution of dextran-based samples at a concentration of 256,000 μg/mL and incubated at 37 °C for 24 h. Antibacterial activity was assessed by measuring the diameter of the inhibition zone around the discs and was expressed as the mean zone of inhibition diameters (mm) produced by dextran. The assay was performed in three replicates for each strain.

### 4.9. Statistical Analysis

All experiments were performed in triplicate, and the results are presented as mean ± standard deviation. Mean values of uracil released from the prepared hydrogels during digestion were statistically evaluated using one-way analysis of variance (ANOVA), followed by Duncan’s test to assess differences between group means (SPSS Statistics 20, IBM Corporation, Armonk, NY, USA). Statistical significance was accepted at *p* < 0.05.

## Figures and Tables

**Table 1:** The levels of uracil released from the prepared hydrogels as a consequence of enzymatic biodegradation occurring in the gastric and intestinal phases.

Uracil-Loaded Hydrogels	Gastric Phase Release, µg/mL	Intestinal Phase Release, µg/mL	Gastric Phase Release, %	Intestinal Phase Release, %	Total Released Amount of Drug, %
Dex-DVB	0	67.81 ± 1.9 ^a^*	0	54.24 ± 1.00 ^a^	54.24 ± 1.00 ^a^
Dex-DEGDA	0	58.69 ± 1.84 ^b^	0	46.95 ± 0.87 ^b^	46.95 ± 0.87 ^b^
Dex-DAAazoB	0	110.66 ± 1.97 ^c^	0	88.52 ± 2.11 ^c^	88.52 ± 2.11 ^c^

* The total amount that can be released is 125 μg/mL, calculated based on 20 mg uracil per g of dry carrier, and 0.25 g of dry material. ^a–c^ values are statistically different from each other.

**Table 2:** Antimicrobial activity of dextran-based samples against Escherichia coli ATCC 8739 and Staphylococcus epidermidis ATCC 12228.

Inhibition Zone (mm)		
Sample Number:	*Escherichia coli* ATCC 8739	*Staphylococcus epidermidis* ATCC 12228
Ampicillin 10 mcg	10	15
Dex-DVB	-	-
Dex-DEGDA	-	-
Dex-DAAazoB	8	7

Legend: “-” no antimicrobial activity. Values are expressed in zone-of-inhibition diameters (mm) and represent the mean value obtained in three independent replicates.

**Table 3:** Initial composition of neat and drug-loaded hydrogels.

Samples	Dex-MA, g	DVB, g	DEGDA, g	DMAAazoN, g	AIBN,g	Uracil, g
Dex-DVB	1	0.1	/	/	0.07	/
Dex-DEGDA	1	/	0.1	/	0.07	/
Dex-DMAAazoB	1	/	/	0.1	0.07	/
Dex-DVB, uracil	1	0.1			0.07	0.02
Dex-DEGDA, uracil	1		0.1		0.07	0.02
Dex-DMAAazoB, uracil	1			0.1	0.07	0.02

## Data Availability

The original contributions presented in this study are included in the article/Supplementary Materials. Further inquiries can be directed to the corresponding author.

## Supplementary Materials

The following supporting information can be downloaded at: https://www.mdpi.com/article/10.3390/gels12010025/s1, Table S1: a. Statistical analysis of the ESR values at pH 3. b. Statistical analysis of the ESR values at pH 6; Table S2: a. Statistical analysis of the mechanical strength values at pH 3. b. Statistical analysis of the mechanical strength values at pH 6; Table S3: Statistical analysis of the digestion of capsulated xerogels in the intestinal phase expressed in concentration and in percent; Table S4: Composition of stock solutions and simulated digestion fluids; Figure S1: FTIR spectrum of neat dextran; Figure S2: FTIR spectrum of uracil.

## Abbreviations

The following abbreviations are used in this manuscript:

DVB	Divinyl benzene
DEGDA	Diethylene glycol diacrylate
DMAAazoB	4,4′-di(methacryloylamino)azobenzene
IARC	International Agency for Research on Cancer
CRC	Colorectal cancer
Dex–MA	Dextran-methacrylate
SEM	Scanning electron microscopy
FTIR	Infrared spectroscopy with Fourier transformation
DSC	Differential scanning calorimetry
DMSO	Dimethyl sulfoxide
SGF	Simulated gastric fluid
