# Supplementary material for: Part II: The Influence of Crosslinking Agents on the Properties and Colon-Targeted Drug Delivery Efficacy of Dextran-Based Hydrogels
Source: Gels. 2025 Dec 28;12(1):25. doi: 10.3390/gels12010025 (PMC12841459; doi:10.3390/gels12010025)
Supplement: Supplementary file 1 [file gels-12-00025-s001.zip › gels-4013390-supplementary.pdf]

Part II: The Influence of Crosslinking Agents on the Properties and Colon-Targeted Drug Delivery Efficacy of Dextran-Based Hydrogels

Tamara Erceg [1,*], Miloš Radosavljević [1], Milorad Miljić [2], Aleksandra Cvetanović Kljakić [1], Sebastian Baloš [3], Katarina Mišković Špoljarić [4], Ivan Ćorić [4], Ljubica Glavaš-Obrovac [4] and Aleksandra Torbica [2]

[1] Faculty of Technology Novi Sad, University of Novi Sad, Bulevar cara Lazara 1, 21000 Novi Sad, Serbia; milosr@tf.uns.ac.rs (M.R.); a.c.istrazivac@gmail.com (A.C.K.)
[2] Institute of Food Technology in Novi Sad, University of Novi Sad, Bulevar cara Lazara 1, 21000 Novi Sad, Serbia; milorad.miljic@fins.uns.ac.rs (M.M.); aleksandra.torbica@fins.uns.ac.rs (A.T.)
[3] Faculty of Technical Sciences, University of Novi Sad, Trg Dositeja Obradovića 6, 21000 Novi Sad, Serbia; sebab@uns.ac.rs
[4] Faculty of Medicine, Josip Juraj Strossmayer University of Osijek, Josipa Huttlera 4, 31000 Osijek, Croatia; kmiskovic@mefos.hr (K.M.Š.); icoric@mefos.hr (I.Ć.); lgobrovac@mefos.hr (L.G.-O.)
* Correspondence: tamara.erceg@uns.ac.rs

Supporting material

FTIR analysis

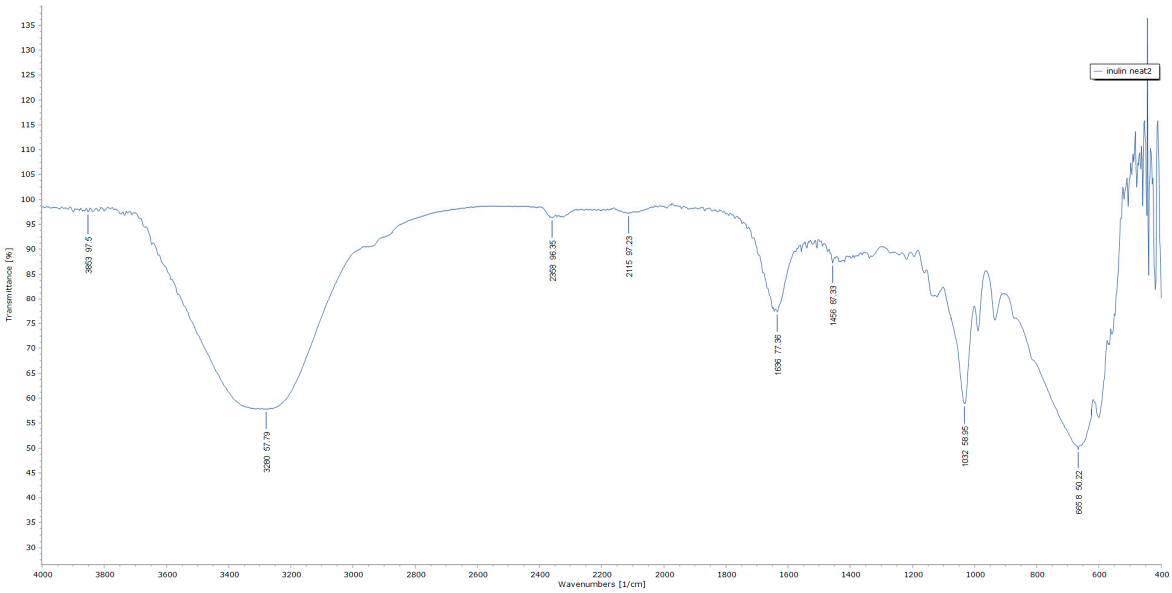

Figure S1. FTIR spectrum of neat dextran.

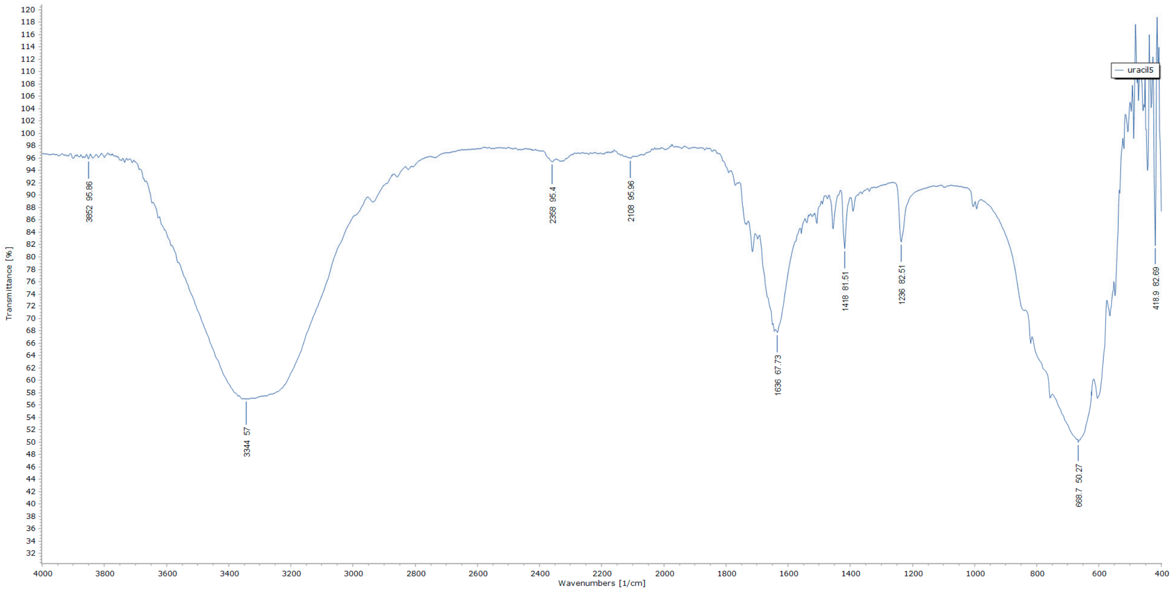

Figure S2. FTIR spectrum of uracil.

Swelling properties

Table S1a. Statistical analysis of the ESR values at pH 3.

Samples	Mean	Median	Standard Error	Standard Deviation	Range	Confidence Level(95.0%)
**Dex-DVB**	1297.04	1296.80	1.81	3.15	6.28	7.81
**Dex-DEGDA**	1994.05	1993.00	2.65	4.61	9.03	11.44
**Dex-DMAAazoB**	2552.91	2553.2	3.19	5.54	11.07	13.76

Table S1b. Statistical analysis of the ESR values at pH 6.

Samples	Mean	Median	Standard Error	Standard Deviation	Range	Confidence Level(95.0%)
**Dex-DVB**	1171.17	1172.14	2.11	3.65	7.1	9.06
**Dex-DEGDA**	1396.03	1394.04	1.99	3.45	5.97	8.56
**Dex-DMAAazoB**	1696.27	1694.32	2.44	4.22	7.74	10.49

Mechanical properties

Table S2a. Statistical analysis of the mechanical strength values at pH 3.

Samples	Mean	Median	Standard Error	Standard Deviation	Range	Confidence Level(95.0%)
**Dex-DVB**	65	64.25	1.28	2.22	4.25	5.52
**Dex-DEGDA**	30	30.25	0.46	0.80	1.55	1.99
**Dex-DMAAazoB**	20	20	0.57	1	2	2.48

Table S2b. Statistical analysis of the mechanical strength values at pH 6.

Samples	Mean	Median	Standard Error	Standard Deviation	Range	Confidence Level(95.0%)
**Dex-DVB**	71	71	0.58	1	2	2.48
**Dex-DEGDA**	55	55	0.29	0.5	1	1.24
**Dex-DMAAazoB**	47	47.5	0.96	1.64	3.25	4.07

Table S3. Statistical analysis of the digestion of capsulated xerogels in the gastric phase expressed in concentration and in percent.

Samples	Mean	Median	Standard Error	Standard Deviation	Range	Confidence Level(95.0%)
**Dex-DVB**	67.81; 54.24	68.39; 54.26	1.10; 0.58	1.10; 1.00	3.68; 2.00	4.74; 2.48
**Dex-DEGDA**	68.69; 46.95	68.69; 46.88	1.06, 0.51	1.84; 0.87	3.67; 1.74	4.65; 2.17
**Dex-DMAAazoB**	110.66; 88.52	110.29; 89.15	1.13; 1.22	1.97; 2.11	3.88; 4.07	4.89; 5.23

Table S4. Composition of stock solutions and simulated digestion fluids.

			**SGF**	**SIF**
			pH 3.0	pH 7.0
**Compound***	Stock conc.		Conc. in SGF	Conc. in SSF
	**g/l**	**mol/l**	**mmol/l**	**mmol/l**
**KCl**	37.3	0.5	6.9	6.8
**KH_2PO_4**	68	0.5	0.9	0.8
**$NaHCO_3$**	84	1	25	85
**NaCl**	117	2	47.2	38.4
**$MgCl_2 \cdot 6H_2O$**	30.5	0.15	0.1	0.33
**$(NH_4)_2CO_3$**	48	0.5	0.5	-
**NaOH**	-	1	-	8.4
**HCl**	-	6	15.6	-
**$CaCl_2 \cdot 2H_2O$**	44.1	0.3	0.075*	0.3

SGF-simulated gastric fluid, SIF- simulated intestinal fluid. All simulated fluids were prepared as 1.25× concentrates since the subsequent addition of enzymes, bile salts, Ca2+ solution, and water will result in the correct concentration of each compound in the final digestion mixture. Concentrations refer to the final digestion mixture; $CaCl_2 \cdot 2H_2O$ was added separately.
